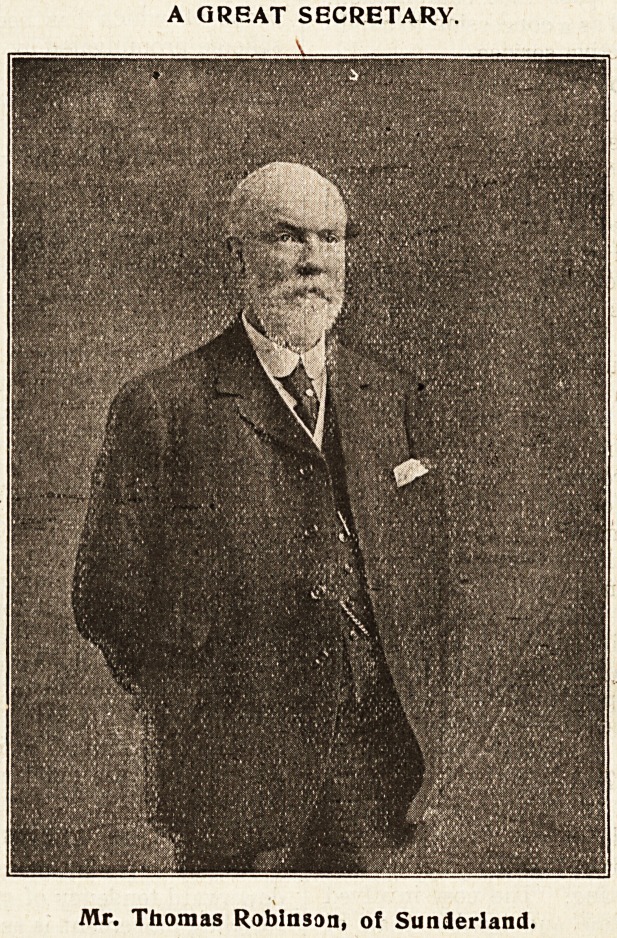# Hospital and Institutional News

**Published:** 1918-11-30

**Authors:** 


### HOSPITAL AND INSTITUTIONAL NEWS.

### A CHEERFUL AMBULANCE.

A HANDSOME motor ambulance has been presented by Mr. S. B. Joel to the Royal Berkshire Hospital. The donor gave a *cachet* to his gift by arriving in the ambulance, fortunately not as a patient, however. Hitherto a horse has drawn the hospital ambulance 2,500 miles a year. Mr. Joel brought the ambulance and the ambulance brought him, a cheerful arrangement which betokened a cheerful giver. The car is a Renault with a Barker body, and is of the type favoured by the Metropolitan Asylums Board.

### RISING SALARIES AT SOUTHAMPTON.

THE Health Committee of the Southampton County Council, which has had the salaries of the health visitors under review, reports that it has found it advisable to improve their positions. The salary is now £100 with £5 extra if with full hospital training, and £5 extra if possessing the certificate of the Sanitary Institute. The committee has now arranged to fix the salary at £110, with the same extra payments, and with an additional £10 to be allotted on the recommendation of the County Medical Officer, thus making a maximum of £130 per annum. The annual allowances of £5 for bicycle and £5 for uniform will remain as before.

### HEALTH CERTIFICATES BEFORE MARRIAGE.

AT a conference recently held in the Town Hall, Eastbourne, and presided over by the Bishop of Norwich, at which Dr. C. W. Saleeby was one of the chief speakers, Miss Martindale, M.D., B.S., in the course of some remarks on venereal disease, urged a system of marriage health certificates. The difficulties of any such system are obvious, yet in many ways something of the kind is highly desirable. It is not only a question of infection of innocent brides and bridegrooms, but the question of the population in the future. For that last reason the question is not only one of venereal disease but of general fitness for parenthood. A certificate that was not narrowed down to the one set of diseases would excite less prejudice than one that certified only freedom from venereal disease. But since no one who suffered from venereal disease in an infectious form could be certified to be fit for fatherhood or motherhood, the matter of innocent infection would be in fact dealt with.

### CERTIFICATES FOR COAL: OFFICIAL WARNING.

WE are asked to state that it has been reported to the Coal Controller that Local Fuel Overseers are being inundated with medical certificates authorising additional coal rations to householders because of illness in the family. Many of these medical certificates have been given, perhaps unwittingly, without the amount of careful consideration which is necessary in view of the present serious coal shortage. The Controller, therefore, draws the attention of medical men to the undesirability of granting these certificates except in cases of real hardship. Otherwise the Controller will be compelled to ignore them altogether. The only condition under which a medical certificate for an extra coal ration on account of illness should be given is where the patient is to remain in bed, and where a fire in the bedroom is necessary for the recovery of the invalid. One hundredweight to one hundredweight and a half a week can be allowed for bedroom fires in these circumstances, and only for the time during which the patient remains in bed.

### SIR COURTAULD THOMSON AND AUSTRIAN WOUNDED.

LORD CAVAN, who was in command of the British Forces in Italy during their recent advance from the Piave, has written to Sir Arthur Stanley, saying: "I wish to write and tell you of the sterling work done by your Chief Commissioner, Sir Courtauld Thomson, during and after the operations in this country. The Austrians abandoned scores of their hospitals, leaving sick and wounded, Italian and Austrian, without food and attention. Many, I fear, have died of starvation and neglect, but the Red Cross and their untiring work have undoubtedly saved many hundreds of lives."

### THE FUTURE OF VOLUNTARY WAR WORKERS.

SIR HORACE MARSHALL, the Lord Mayor, is concerned in regard to the future of voluntary war workers, and asks what is to become of them when their services shall be no longer required. He sees a great opportunity to organise this voluntary effort and to continue it for social service, and hopes that since it is largely of local origin the existing organisations may not be dispersed before some attempt has been made to convert them to other social uses. The war is a cry to which all turn. Therefore the chiefs of all local centres should be asked by the Director of Voluntary Organisations not to disband their workers. He should also make it his business to supply information as to how they can best be used. The disabled, the pensioners, will need attention for a long time, and the Director should make it his business to suggest a policy for the future.

### TO BE DISCONTINUED IN OUR NEXT.

As we turn over the pages of the *Gazette* of the 3rd London General Hospital, a pleasant excitement now begins to be felt. One expects to read the anticipatory announcement of the last number. Since the writers of that entertaining periodical have often looked forward to its extinction, its admirers may do so without fear of being misunderstood. Still Donovan, who has been a joy to many during the war, would hardly claim to be a joy *for ever*. Songs to our Sisters are often pleasant reading, like rhyming alphabets, heard about hospitals and the like; there is a time, as the prophet said, to refrain from them. It has all been, it is still, very good fun, and we hope that its readers and its writers will carry into journalism something of the standard which they have maintained in the most humorous and best illustrated of the English hospital journals. For the last number, when it shall come, Noel

Irving, J. H. Dowd, R. B. Ogle, G. J. Coates, and Miss H. M. Nightingale should let themselves go. We hope they will.

### THE PROVISION OF TEMPORARY LIMBS.

MANY medical men will have amongst their patients men who have lost a leg in the war and who either have not been provided with a permanent artificial leg or who have an artificial leg that for some reason or other does not fit. It may be useful to such to know that temporary limbs can be obtained through the Joint War Committee of the British Red Cross and Order of St. John through the depôts recently set up or taken over by the committee. Unfortunately, instead of one authority to whom to apply there seem to be several, differing according as the patient is a soldier on furlough, or a pensioner or a man in hospital. It seems, however, that a good rule for a busy doctor will be: apply to the Local War Pensions Committee, or direct to Provisional Limbs Department, 83 Pall Mall, S.W. 1.

### MORE GLYCERIN FOR MEDICAL PURPOSES.

MAINLY through the efforts of the Pharmaceutical Society of Great Britain, there is a probability that a supply of glycerin will be released for *strictly medical purposes* in the near future. This will come as welcome news to medical men and dispensers, as, whilst substitutes have been invented and are suitable for certain purposes, no really good substitute for glycerin has been discovered which acts in the same way when used on tampons, for the throat, etc. It is also a necessary drug for the preparing of glycerophosphates, which are largely prescribed by medical practitioners. The release of glycerin will be granted by the Ministry of Munitions, who will probably ask the Pharmaceutical Society to allocate a proportion to those chemists and hospitals who apply. No doubt the armistice situation will ease the demand for explosives, and consequently glycerin. As regards drugs and chemicals, it would seem prudent not to buy these in large quantities at the high prices now ruling.

### CLASSIFICATION BY INSTITUTION.

THERE have been many criticisms of Mr. Dawson's plan for reorganising the hospitals of Bradford on municipal lines by handing over St. Luke's from the Guardians to the City Council, and thereby, as he believed, avoiding the need for a new general voluntary hospital. It is interesting that THE HOSPITAL's criticisms have been succeeded by those of Mr. F. H. Bentham, who, as an ex-chairman of the Bradford Board of Guardians, has experience similar to that of Mr. Dawson himself. Mr. Bentham gives careful and extended reasons for thinking that a new great hospital should be built for the city; and the proposed new Royal Infirmary finds in him a judicious champion. He has pointed out, in an admirable statement given to the *Yorkshire Observer*, the right of admission to a municipal hospital necessitates "restrictions upon the person admitted" which become irksome. It is, he says, these conditions which have led Poor-Law relief to be regarded as involving a stigma. Mr. Bentham, too, prefers classification by institutions rather than by separate wards in the same building. His statement is full of points which deserve careful attention.

### A POLICY FOR THE PRINCE OF WALES' FUND.

SINCE only two-thirds of the vast sum entrusted to the Prince of Wales' Fund has yet been spent, and some three millions remain in hand, the committee of the Fund should state their proposals for spending the remainder. The money was given to be spent to alleviate distress arising from the war, and this fact should rule out at once the eager claims of individual institutions to participate in it. If the committee has a policy it should state it. If not, as is to be feared, it should be convened to decide one. Its aim should be, however, not to "pauperise" Government Departments by relieving them of necessary expenditure. For instance, it should not attempt to do the work of the Ministry of Pensions. Its aim should be, as was intended originally, to look after those who were being neglected by every other body. No old-established institution or fund should benefit by it. It should alleviate those kinds of distress arising from the war for which no provision has been made. It should also be careful to spend its money: that is what its money is for. With these two principles, care and thought alone are required to define the scope of its benevolence.

### CARDINAL BOURNE ON THE NURSING VOCATION.

THE ladies of the Catholic Nursing Guild listened recently to an interesting and inspiring address by Cardinal Bourne on the nursing vocation. The address was delivered in the chapel of the Convent of the Cenacle, Stamford Hill. After emphasising the importance which during the war had attached to the nursing body throughout the world, his Eminence said that hundreds and thousands of women had fitted themselves to engage in that great work of mercy. Many of them, having discovered that this was their true vocation, would continue that work after the war, and in that number there was bound to be a considerable body of Catholic nurses. He added: "The work of nursing is one for which Our Divine Master has promised an immense reward, on condition that we see Him in all the sick and wounded to whom we minister. In other words, you are to take a supernatural view of the work to which you are called. To a certain extent, self-devotion, inspired by the supernatural, does make up for a certain want of technical skill. The value of any nurse, no matter how highly trained, no matter how technically skilful she may be, is enormously enhanced if in addition to that skill she is leading a life closely united to Almighty God and looking always at her work from a supernatural standpoint. That is the great appeal which the Guild has to make to Catholic nurses in order to get them to realise, individually and as a body, that their work is a great spiritual work, and one that can only be accomplished perfectly if they are united to God, looking to Him for His blessing on their work."

### A HOSPITAL SECRETARY TO EMULATE.

WHEN Mr. Thomas Robinson died, aged sixty-eight, on the 31st ultimo, the Royal Infirmary, Sunderland, lost a secretary who had served it faithfully for thirty-six years. His sudden death was without warning, save for the cardiac seizure which occurred three months ago. He complained of feeling unwell at the station to which he had gone in search of a cab, after attending a meeting at the infirmary at 5.40 P.M., and it was within an hour in the waiting-room before a doctor could be fetched that he died. Since the Coroner happened to have been with Mr. Robinson less than an hour before his death, an inquest was deemed unnecessary. The son of the then stationmaster at Hylton, after some experience in an industrial works at Washington, Mr. Robinson became secretary to the Sunderland Infirmary in 1882. Since then the beds have increased from 96 to 236, the in-patients from 1,160 to 3,000, and the income from £6,350 to over £24,000. There were some workmen's donations in the 'eighties, but Mr. Robinson is credited with the success with which they have been developed. The chief extensions during his term of office were the Edward Backhouse Wing, which was opened in 1882 with forty-four beds, and the James Hartley Wing, opened in 1889 with sixty-eight beds. From the town's Jubilee Fund a Nurses' Home was built in 1898. Two years later the Ladies' Guild was founded, and we must not forget the Heatherdene Convalescent Home, opened in 1892. In 1911 the King granted the hospital the title of "Royal," and a year later a children's hospital was opened with fifty-four beds, to be increased eventually to sixty-six. Mr. Robinson's life was interweaved so closely with that of his institution that a chronicle of its activities is really, in his case, the story of his own. He was much esteemed. His widow, Mrs. Robinson, has received many signs of sympathy. Of her three sons and one daughter, the latter is married to the Harbour Master of Bombay, the eldest son is an engineer in India, and the youngest died in Mesopotamia. A third son is at Coventry.

A GREAT SECRETARY.

Mr. Thomas Robinson, of Sunderland.

### CHAOS IN BERMONDSEY: A RESOLUTION RESCINDED.

BERMONDSEY Board of Guardians have rescinded the resolution recorded in last week's issue of THE HOSPITAL, page 153, with reference to the purchases of large stocks of drapery by their clerk (Mr. Pitts Fenton). The resolution appeared to have got the Guardians out of a difficulty, for it not only protested against the charges against them of being lax in their administration, which they hold are unjustifiable, but it also assisted in saving the clerk from the consequences of any drastic resolution which might lead to him forfeiting his pension. By ten votes to nine the Guardians rescinded this resolution, the minority containing two members who had not attended a board or committee meeting for seven months, whilst a third had not been present for close on two years. The board decided to leave the decision on the clerk to the Local Government Board, at the same time informing them that they had lost their confidence in him. The next move rests with the authorities at Whitehall, but, whatever may be their decision, it is apparent that matters cannot be allowed to go on as they have done in Bermondsey. There appears to be not only a great lack of confidence, but practically the ignoring of each other by the clerk and the Guardians. This must lead to chaos, and to further bad administration. The Local Government Board should take a strong attitude in bringing this unfortunate situation to a speedy termination in the interests of all concerned.

### PHARMACEUTICAL EXAMINATION CHANGES.

Two important changes in the regulations governing the admission of pharmacists to full qualification have been decided upon by the council of the Pharmaceutical Society. Both these changes will make it easier for students to obtain the qualification, and at the same time the safeguards ensuring sufficient training and knowledge will be adequate. In the past it has been necessary for candidates for the final examination to pass in all the subjects at the same examination. The council has, however, adopted the recommendation of its Reconstruction Committee that, if a candidate does not pass in all the subjects at one and the same examination, he should be excused from taking the subjects in which he has passed at a subsequent examination. The other change is a relaxation of the routine requirements for the acceptance of certificates in connection with the preliminary examination. A special committee is to be empowered to admit to registration as students applicants possessing a standard of preliminary knowledge to justify the belief that they would be competent, after the necessary course of training in the subjects of the qualifying examination, to pass that examination. Presumably this is intended as a concession to candidates who have been on active service.

### THE APPEAL OF THE MAP.

The war has done something to teach us geography—the most neglected of subjects in schools—by means of maps. As a means of appeal, therefore, the hospital map has something to recommend it, and it has been employed by the Great Northern Central Hospital. Printed in colours, and in themselves, the map forms the outside of a leaflet which contains within those facts and figures represented cartographically without. The streets, like a mesh of veins in, technically, a putrid blue, fade away into green spaces in which such names as Enfield, Bush Hill, and Hadley Woods are inscribed. But the centre of this livid picture is the brilliantly lit building in the foreground, which is the Great Northern Central Hospital. Of course the representation is a map mainly in name: it does not profess to vie with the masterpieces of Mr. Bartholomew. It gives an immediate idea of the size of the area which the hospital serves, but the reader who loves maps will not be able to peruse it in detail. That maps can be read with the same pleasure as other forms of printed books we remember to have been convinced by an article which appeared, if we remember, in January or February 1911 in the now defunct *Tramp*. Our only point is that if any secretary is in the habit of reading maps for pleasure he could devise a hospital map which would serve the purpose of a broadside and yet be intrinsically interesting. The cost involved would rather be that of time and trouble than of printing.

### £10,000 FOR WALSALL GENERAL HOSPITAL.

The committee of the Walsall General Hospital have great pleasure in announcing that the sum of £10,000 for which they have appealed to the public of Walsall and district has now been raised. The appeal was inaugurated at a special meeting held at the Council House on June 19, and it is a source of much satisfaction to the committee to be able to report its completion in less than five months from that date. The Mayor of Walsall, Councillor S. M. Slater, has taken a deep interest in the scheme, and his efforts have had much to do with its success. The committee are much gratified to think that the desired result should have been attained during the Mayoralty of Mr. Slater, and, in offering him their congratulations and hearty thanks, they feel that he will share their pleasure in its accomplishment. The committee desire to take the opportunity of thanking all those who have helped in the special appeal, and they trust that those who have not yet contributed will take an early opportunity of doing so. The advance of medical and surgical science has been so rapid during the last few years that a very large sum is needed to keep the hospital abreast of the times, and the committee have good hope that when the fund is finally closed the total amount will greatly exceed the £10,000 originally asked for.

### APPOINTMENTS OLD AND NEW.

The ancient Sherburn Hospital, a charity the scope of which has been widely increased by a scheme promulgated by the Charity Commissioners, is seeking the services of a clerk at £250 a year, with provision in regard to offices, and the attention of disabled officers and men of His Majesty's Forces is called to the vacancy. Something of the history and recent developments of this charity has been alluded to in The Hospital (September 21, 1918, page 530, and September 28, 1918, page 549), and it is evident that the increased usefulness of the hospital will provide interesting work for the new clerk or secretary. In looking through copies of some of the well-known Egerton Papers a few days ago, we noticed amongst these documents, accumulated by Sir Thomas Egerton, Keeper to the Great Seal to Queen Elizabeth and later Lord Chancellor under James I., a letter in reference to Dr. Valentine Dale, who was collated to the Mastership of Sherburn Hospital on March 22, 1584. The Queen's Secretary in this document states that "Hir Majestie beeing moved lately touching Mr. Doctor Dale his byll for his ryght of presentation in the hospitall of Sherburne, is gratiously contented to sygne the same." Dr. Dale's duties did not tie him constantly to the district of Durham, for in Surtees' "History of Durham" it is recorded that he was sent to expostulate with the Prince of Parma upon the publication of a tract by Cardinal Allen.

### THIS WEEK'S DRUG MARKET.

As we have intimated would be the case, the downward tendency of prices has begun. True, the receding movement is as yet slow, but it may gather impetus as it goes on. It is, of course, a gratifying fact that the fall in prices has not been sudden or far, since it is obviously undesirable that there should be anything in the nature of a panic. Fortunately stocks, such as they are, appear to be in strong hands—a fact which checks, or at any rate tends to check, any rapid downward movement in values. Among the articles which may be cited as being definitely lower in price are sugar of milk, Japanese peppermint oil, clove oil, saccharin, antifebrin, benzoic acid, and salicylic acid, while the number of drugs that have an "easier" tendency is much larger. Wise buyers will continue to act with special caution, and will not increase their stocks beyond what is necessary to carry them over a short period.

## Figures and Tables

**Figure f1:**